# RNase L is a negative regulator of cell migration

**DOI:** 10.18632/oncotarget.6246

**Published:** 2015-10-27

**Authors:** Shuvojit Banerjee, Geqiang Li, Yize Li, Christina Gaughan, Danika Baskar, Yvonne Parker, Daniel J. Lindner, Susan R. Weiss, Robert H. Silverman

**Affiliations:** ^1^ Department of Cancer Biology, Lerner Research Institute, Cleveland Clinic, Cleveland, Ohio, USA; ^2^ Department of Microbiology, Perelman School of Medicine, University of Pennsylvania, Philadelphia, Pennsylvania, USA; ^3^ Translational Hematology and Oncology Research, Taussig Cancer Institute, Lerner Research Institute, Cleveland Clinic, Cleveland, Ohio, USA

**Keywords:** RNase L, migration, metastasis, FAK, prostate cancer

## Abstract

RNase L is a regulated endoribonuclease that functions in the interferon antiviral response. Activation of RNase L by 2′, 5′-oligoadenylates has been linked to apoptosis, autophagy and inflammation. Genetic studies have also suggested the possible involvement of the RNase L gene (*RNASEL*) on chromosome 1q25.3 in several types of cancer. Here we report that ablation of RNase L in human prostate cancer PC3 cells by CRISPR/Cas9 gene editing technology enhanced cell migration as determined both by transwell assays and scratch wound healing assays. In addition, RNase L knockdown by means of RNAi increased migration of PC3 and DU145 cells in response to either fibronectin or serum stimulation, as did homozygous disruption of the RNase L gene in mouse embryonic fibroblasts. Serum or fibronectin stimulation of focal adhesion kinase (FAK) autophosphorylation on tyrosine-397 was increased by either knockdown or ablation of RNase L. In contrast, a missense mutant RNase L (R667A) lacking catalytic activity failed to suppress cell migration in PC3 cells. However, a nuclease-inactive mutant mouse RNase L (W630A) was able to partially inhibit migration of mouse fibroblasts. Consistent with a role for the catalytic activity of RNase L, transfection of PC3 cells with the RNase L activator, 2′, 5′-oligoadenylate, suppressed cell migration. RNase L knockdown in PC3 cells enhanced tumor growth and metastasis following implantation in the mouse prostate. Our results suggest that naturally occurring mutations in the RNase L gene might promote enhanced cell migration and metastasis.

## INTRODUCTION

The 2′, 5′-oligoadenylate (2–5A) synthetase (OAS)-RNase L pathway is one of the principal components of antiviral innate immunity in higher vertebrates [1, 2]. In addition, some genetic studies have suggested a wider role for RNase L beyond the interferon (IFN) induced antiviral state. In particular, a combined positional cloning and candidate gene approach identified the RNase L gene (*RNASEL*) as a candidate for the hereditary prostate cancer 1 (HPC1) locus at human chromosome 1q24–25 [3]. Since then numerous and sometimes conflicting reports either found an association, e.g. [4–6] or no association, e.g. [7–9] of *RNASEL* mutations and variants with hereditary or sporadic prostate cancer. The varied findings on *RNASEL* and prostate cancer could be due to differences between patient populations or exposure to environmental agents such as infection [10]. However, while still a possibility, there is presently no compelling evidence for involvement of viral infections in prostate cancer. Mutations in *RNASEL* have also been linked to risk other types of cancer, including head and neck, uterine, cervix, breast [11], pancreatic [12] and hereditary non-polyposis colorectal cancer [13]. Additional activities of RNase L beyond its antiviral activity include suppression of the mobile genetic element LINE-1 [14] or stimulation of apoptosis [15, 16], inflammation [17], and autophagy [18, 19], any one of which could potentially affect cancer development.

RNase L is activated by 2–5A [mainly p_3_5′(A2′p5′)_2_A] produced from ATP in response to stimulation of OAS enzymes by viral double-stranded (ds) RNA [2, 20]. However, some cellular RNAs are also capable of activating OAS, albeit weakly compared with viral dsRNA. For instance, we reported that prostate cancer cell lines (PC3, LNCaP and DU145) expressed higher levels of RNA molecules capable of binding and activating OAS then did normal prostate epithelial cells (PrEC) [21]. These OAS activators were identified as mRNAs for Raf kinase inhibitor protein (RKIP) and poly(rC)-binding protein2 (PCBP2) and human endogenous retrovirus (hERV) envelope RNAs. In the same study, PCBP2 mRNA was also found to be elevated in metastatic prostate cancer tissues.

To study if RNase L has a role in cell migration, here we investigated the effect of RNase L on the migration of prostate cancer cells, as well as mouse embryonic fibroblasts (MEF). Our findings show that ablation or knockdown of RNase L enhanced the migration of both human prostate cancer cells and of MEF, raising the possibility that *RNASEL* mutations might contribute to metastasis.

## RESULTS

### CRISPR/Cas9 disruption of the RNase L gene enhances the migration of human prostate cancer PC3 cells

**Figure 1:**
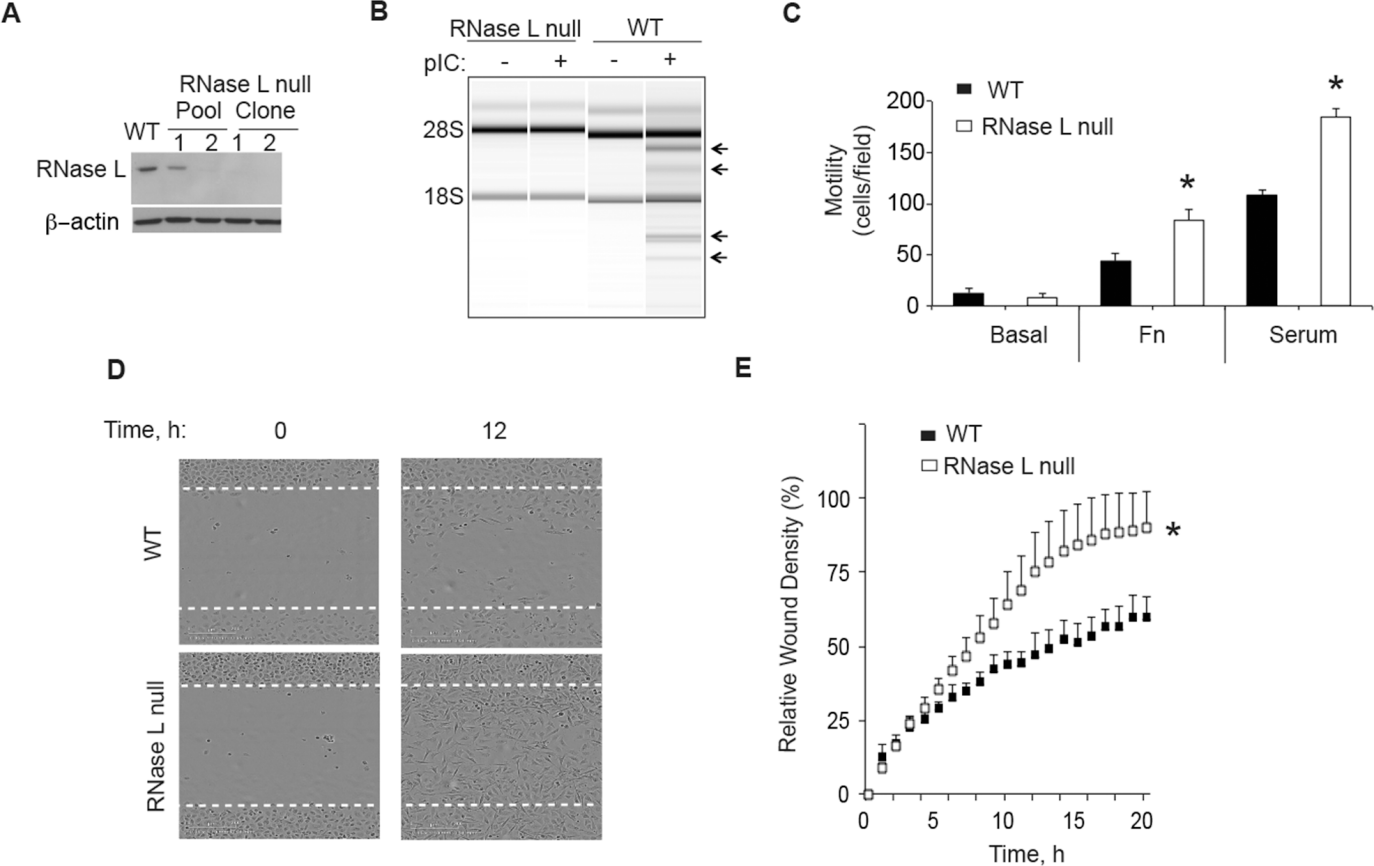
CRISP/Cas9 ablation of RNase L enhances PC3 cell migration (**A**) RNase L and β-actin levels determined by immunoblotting extracts of PC3 WT cells, PC3 pools infected with lentivirus CRISP/Cas9 construct targeting the RNase L gene, and two drug-selected clones from pool 2. (**B**) RNase L mediated cleavage of rRNA as determined in RNA chips (Agilent) in response to pIC transfection of PC3 RNase L-null clone 1 (cl1) or PC3 WT cells. (**C**) Migration of PC3 WT and RNase L-null cells (cl1) through transwell membranes towards BSA (basal), fibronectin (FN) or serum were determined by cell staining and counting from three fields/replicate. Data are shown as the mean ± standard deviation (SD) from a single experiment with 3 technical replicates. *p < 0.05 by Student's two-tailed *t* tests. The experiment was conducted times (biological replicates) with similar results. (**D**) (**E**) Scratch wound healing assays of PC3 WT cells or PC3 RNase L-null cl1 cells. Data in panel E are shown as the mean ± SD from a single experiment with 6 technical replicates. *p < 0.05 by Student's two-tailed *t* tests. The experiment was conducted times (biological replicates) with similar results.

To determine the effect of RNase L on cell migration, RNase L was ablated in PC3 cells using CRISPR/Cas9 gene editing technology. There was no detectable RNase L in PC3 cells containing the CRISPR/Cas9 construct targeting the RNase L gene, as determined by Western blotting two clonal cell lines, including clonal cell line PC3-cl1 used for these experiments (Figure 1A). The absence of RNase L in these cells was validated by a functional assay in which the synthetic dsRNA, poly(I):poly(C) (pIC), an activator of 2′, 5′-oligoadenylate synthetases (OAS), was transfected followed by isolation and separation of total RNA on RNA chips (Agilent). OAS enzymes produce the 2′, 5′-oligoadenylate activators (2–5A) of RNase L from ATP in response to stimulation by dsRNA [20]. Specific and characteristic RNase L-mediated cleavage of rRNA [22, 23] was observed in the pIC transfected control cells, but not in the CRISPR/Cas9 RNase L knockout cells (Figure 1B). The RNase L-mediated cleavage products of 28S and 18S rRNA were previously established by Northern blot analysis with radiolabeled 28S and 18S cDNA [22]. Cell migration was then measured in transwell haptotaxis migration assays by placing cells in the upper chamber and either fibronectin or serum in the lower chamber. Following an incubation period of 4 h, the cells that migrated through the membrane were stained and counted. The control PC3 cells and RNase L-null PC3-cl1 cells showed only low basal levels of cell migration (Figure 1C). In contrast, cell migration was greatly increased in response to either fibronectin or serum. Furthermore, migration of RNase L-null PC3-cl1 cells in response to fibronectin or serum was increased by 90% and 70%, respectively, compared to the control PC3 cells. To confirm the effect of RNase L ablation on cell migration, scratch wound healing assays were performed. After 24 h of serum stimulation, total wound closure was increased by 47% in the RNase L-null PC3-cl1 cells compared to the control cells, as determined by IncuCyte ZOOM^®^ Live Cell Imaging (Figure 1D, 1E). In contrast, there was no significant difference in cell proliferation between these two cells lines with up to72 h of serum stimulation (data not shown). These results show that ablation of RNase L in PC3 cells greatly enhanced their migration, likely by decreasing adhesion to the extracellular matrix or otherwise increasing cell motility.

### Depletion of RNase L levels by RNAi enhances migration of PC3 prostate cancer cells

**Figure 2:**
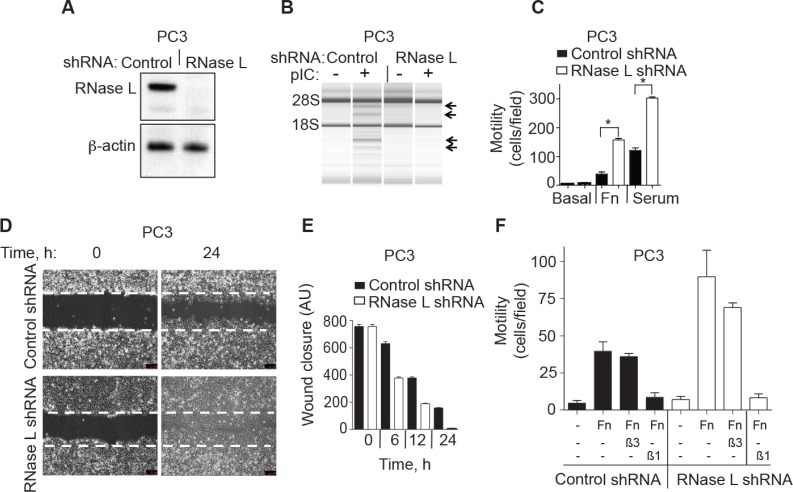
RNase L depletion by RNAi increases migration of PC3 cells (**A**) RNase L and β-actin levels in PC3 cells stably expressing control or RNase L shRNAs as determined in immunoblots. (**B**) RNase L activity determined by rRNA cleavage in response to pIC transfection monitored in RNA chips (Agilent). (**C**−**E**) Migration of PC3 control shRNA- and RNase L shRNA-expressing cells by either (C) transwell assays or by (D, E) scratch wound healing assays. AU, arbitrary units. (**F**) Cells were pre-treated with blocking integrin β1 (β1) or integrin alpha V β3 (β3) antibodies for 30 min before plating into transwells measuring migration in response to FN. C-F Data are shown as the mean ± SD each from a single experiment with 3 technical replicates. *p < 0.05 by Student's two-tailed *t* tests. The experiments were conducted 3 times (biological replicates) with similar results.

To confirm the effect of RNase L on cell migration, stable expression of a short hairpin (shRNA) was used to deplete RNase L levels in PC3 cells (Figure 2). Efficient knockdown of RNase L in PC3 cells stably-expressing shRNA against RNase L mRNA was shown by immunoblotting (Figure 2A). There was no significant difference in the cell growth rates between the control and RNase L shRNA cells up to 72 h (data not shown). RNase L activity in the intact cells was monitored by rRNA cleavage assays. Following pIC transfection there was characteristic RNase L-mediated cleavage in rRNA in control cells but not in the cells stably expressing shRNA against RNase L mRNA (Figure 2B). In transwell assays, migration of the RNase L knockdown cell lines in response to either fibronectin or serum was increased by 250% and 114%, respectively, compared to the control cells (Figure 2C). These findings were extended by manual scratch assays that showed that wound closure by 12 h was increased by 90% in the RNase L shRNA cells compared with control cells (Figure 2D, 2E). Therefore, in PC3 cells either ablation of RNase L by CRISPR/Cas9 or depletion of RNase L by means of shRNA had the same effect of greatly enhancing cell migration.

To further characterize the inhibitory role of RNase L on cell migration, both control and RNase L shRNA PC3 cells were pre-incubated with functional blocking antibodies to integrin β1 or integrin alpha Vβ3. In transwell assays, antibody against integrin β1, but not integrin alpha Vβ3, prevented cell migration in response to fibronectin in both cell types (Figure 2F).

### RNase L restricts the migration of human prostate cancer DU145 cells

**Figure 3:**
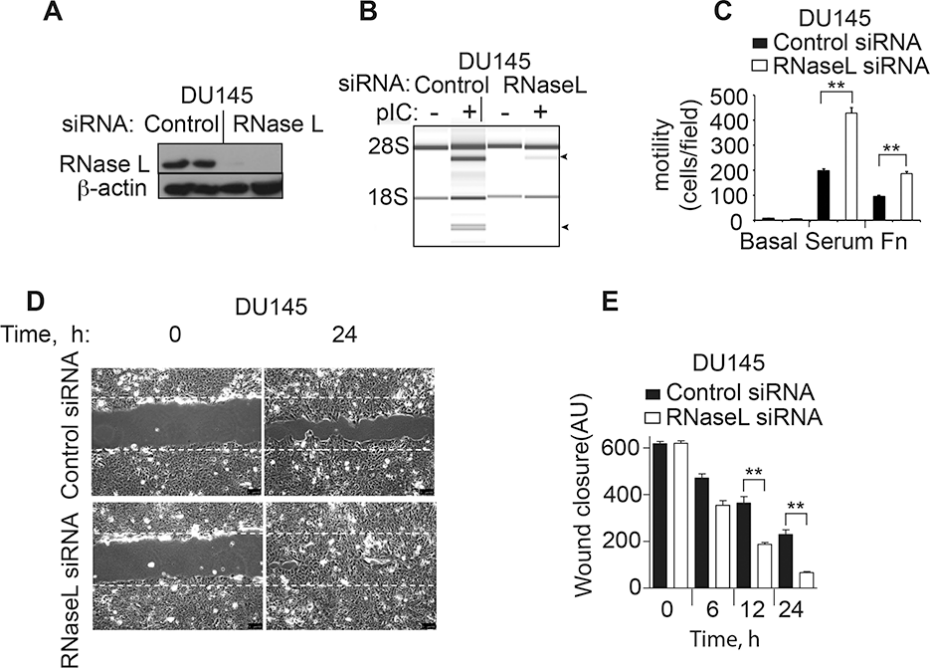
RNase L depletion by RNAi increases migration of DU145 cells (**A**) RNase L and β-actin levels in DU145 cells transfected with control or RNase L siRNA oligonucleotides as determined in immunoblots. (**B**) RNase L activity determined by rRNA cleavage in response to pIC transfection monitored in RNA chips (Agilent). (**C**−**E**) Migration of DU145 control shRNA- and RNase L shRNA-expressing cells by either (C) transwell assays or by (D, E) scratch wound healing assays. AU, arbitrary units. C–E. Data are shown as the mean ± SD each from a single experiment with 3 technical replicates. **p < 0.001 by Student's two-tailed *t* tests. The experiments were conducted 3 times (biological replicates) with similar results.

To extend these results to another prostate cancer cell line, DU145 cells were examined. SiRNA oligonucleotides were used to transiently deplete RNase L in the DU145 cells. Immunoblotting was used to monitor RNase L levels in DU145 cells at 48 h after transfection with either a control siRNA or siRNA against RNase L (Figure 3A). RNase L was reduced to undetectable levels by the siRNA treatment. Furthermore, RNase L cleavage of rRNA in response to pIC was prevented by the siRNA against RNase L mRNA (Figure 3B). Depletion of RNase L levels enhanced migration in transwell assays by 118% and 95% in response to serum or fibronectin, respectively, in comparison to the control cells (Figure 3C). Finally, wound closure by manual scratch assays was enhanced by 180% at 24 h in the DU145 cells treated with siRNA against RNase L mRNA compared to the cells treated with the control oligonucleotide (Figure 3D, 3E). Taken together, our results show an inhibitory effect of RNase L on cell migration in two different prostate cancer cell lines, PC3 and DU145 cells.

### Targeted disruption of the RNase L gene in mouse embryonic fibroblasts (MEF) enhances migration, whereas over-expression of RNase L reduces migration

**Figure 4:**
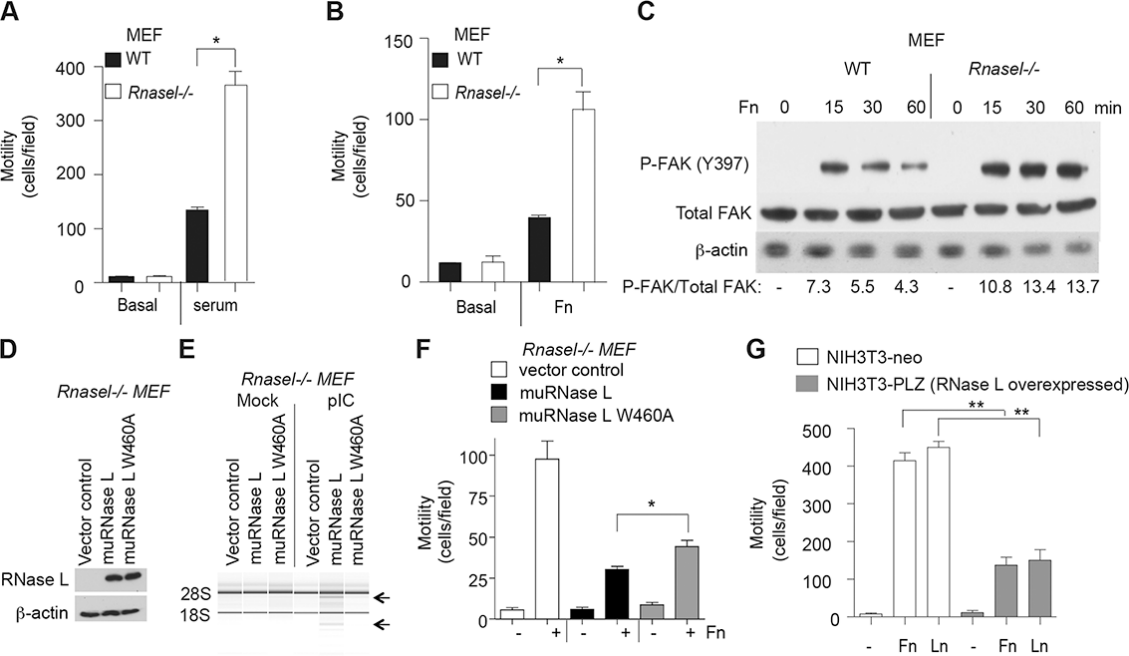
Suppression of mouse fibroblast migration by RNase L (**A, B**) Transwell assays showing migration of WT MEF and *Rnasel−/−* MEF in response to (A) serum or (B) fibronectin (FN). (**C**) Levels of FAK phosphorylated on tyrosine 397, total FAK, and β-actin levels from WT MEF and *Rnasel−/−* MEF after plating on dishes coated with FN for different times (as indicated) determined by immunoblotting. (**D**) Expression of flag-tagged mouse WT RNase L cDNA and mouse mutant RNase L W630A cDNA in *Rnasel−/−* MEF as determined by immunoblotting with anti-FLAG antibody. (**E**) RNA integrity before and after pIC transfection as determined in an RNA chip (Agilent). (**F**) Cell migration in transwell assays in response to fibronectin (Fn). (**G**) Migration of NIH3T3-neo and NIH3T3-PLZ cells over-expressing human RNase L in the presence of FN or laminin (LN). A,B,F,G. Data are shown as the mean ± SD each from a single experiment with 3 technical replicates. *p < 0.05, **p < 0.001by Student's two-tailed *t* tests. The experiments were conducted 3 times (biological replicates) with similar results.

To determine if the inhibitory effect of RNase L on cell migration extended to mouse cells, we studied wild type (WT) and *Rnasel*−/− MEF. In absence of added serum or extracellular matrix proteins, WT and *Rnasel*−/− MEFs were unable to migrate in transwells (Figure 4A, 4B). In contrast, cell migration occurred in response to either serum or fibronectin. Migration of the *Rnasel*−/− MEF was enhanced by about 192% and 100% in response to serum or fibronectin, respectively, compared with WT MEF (Figure 4A, 4B).

Integrin receptor clustering in response to extracellular matrix promotes activation of focal adhesion kinase (FAK), a cytoplasmic tyrosine kinase with roles in cell adhesion, migration and survival (reviewed in [24, 25]). When activated, FAK undergoes a conformational change leading to autophosphorylation on tyrosine-397, leading to phosphorylation at other sites by src kinases which increase FAK activity. Stimulation of FAK autophosphorylation in response to fibronectin for 15 min was increased by 48% in *Rnasel*−/− MEF compared with WT MEF (Figure 4C). In addition, FAK phosphorylation at this site remained elevated for longer in the *Rnasel*−/− MEF (compare the 60 min lanes for the WT and *Rnasel*−/− MEF). These results show that the effect of RNase L on cell migration is mediated at an early step in the signaling pathway, at or before FAK autophosphorylation.

To determine if the catalytic domain of RNase L was required for suppression of cell migration, the *Rnasel*−/− MEF were transiently transfected with either WT mouse RNase L, or a nuclease-inactive mutant mouse RNase L W630A [26] (Figure 4D). Transfection with pIC resulted in activation of WT RNase L but not the mutant as determined by rRNA cleavage assays (Figure 4E). Both WT and mutant RNase L suppressed cell migration in response to fibronectin stimulation as determined in transwell assays (Figure 4F). However, WT RNase L produced greater inhibition of migration (71%) than the mutant (46%). These findings suggest that the catalytic function of RNase L is required for optimal inhibition of cell migration.

In contrast to findings on RNase L deficient cells, over-expression of RNase L by (∼100-fold) in mouse NIH3T3 fibroblasts [27] reduced migration by 67% and 66% in response to fibronectin or laminin, respectively (Figure 4G). These findings confirm that RNase L restricts the migration of mouse as well as human cells.

### The catalytic domain of RNase L contributes to inhibition of cell migration

**Figure 5:**
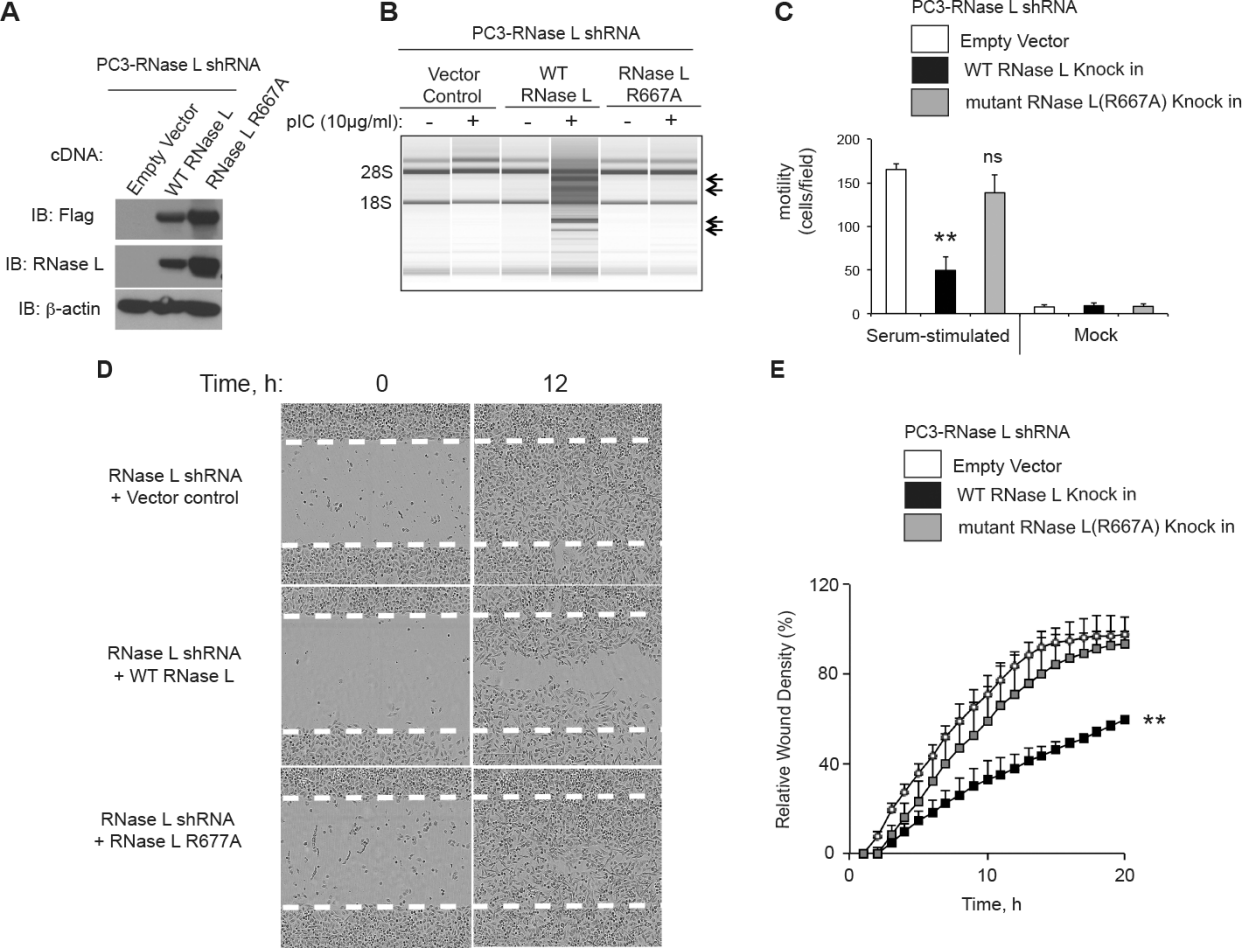
The catalytic domain of RNase L is required for inhibition of PC3 cell migration (**A**) Levels of Flag-tagged WT RNase L, Flag-tagged R667A mutant RNase L and β-actin in immunoblots of extracts of PC3 cells stably-expressing shRNA against RNase L and infected with the indicated cDNAs in lentiviruses followed by drug selection. Immunoblots were probed with antibodies against the Flag epitope (top), monoclonal antibody against human RNase L (middle), and against β-actin (bottom). (**B**) RNA chip results (Agilent) of PC3 cells (as indicated) that were mock transfected or transfected with pIC. (**C**) Cell migration in transwells in response to serum as compared with BSA (mock). (**D, E**) Scratch wound healing assays of PC3 cells in which RNase L was depleted with shRNA and then transfected with empty vector, WT RNase L, or mutant R668A RNase L cDNAs (as indicated). (C−E) Data are shown as the mean ± SD each from a single experiment with (C) 3 and (E) 6 technical replicates. **p < 0.001by Student's two-tailed *t* tests. The experiments were conducted 3 times (biological replicates) with similar results.

To determine if the catalytic domain of RNase L is involved in controlling cell migration, RNase L was stably depleted in PC3 cells with shRNA against the 3′-UTR of RNase L mRNA followed by drug selection. The efficacy of shRNA against RNase L is shown in Western blots probed with monoclonal antibody to RNase L (Figure 2A, 5A). Subsequently, cells were stably reconstituted with cDNA for either WT or mutant RNase L (R667A) lacking nuclease activity [26]. Because the WT and R667A mutant cDNAs lacked the natural 3′-UTR sequence they were expressed normally in the presence of the shRNA (Figure 5A). RNase L activity was determined by rRNA cleavage in response to pIC transfections (Figure 5B). WT RNase L produced extensive rRNA cleavage, whereas there was no detectable degradation in rRNA in the PC3-RNase L shRNA cells containing the vector or expressing mutant RNase L R667A.

Surprisingly, only the WT RNase L, and not its inactive mutant R667A, suppressed migration in response to serum as determined in transwell assays (Figure 5C). This was despite higher levels of mutant RNase L compared to WT RNase L (Figure 5A). These findings were confirmed in scratch assays with IncuCyte ZOOM^®^ live cell imaging (Figure 5E, 5F). Again, only WT RNase L inhibited cell migration when compared to the empty vector control. There was a 40% reduction in wound healing at 20 h in the WT RNase L expressing cells compared with empty vector or mutant RNase L expressing cells. These results suggest that the catalytic domain is essential for the inhibition of cell migration by RNase L in PC3 cells.

### Direct activation of RNase L by 2-5A suppresses cell migration

**Figure 6:**
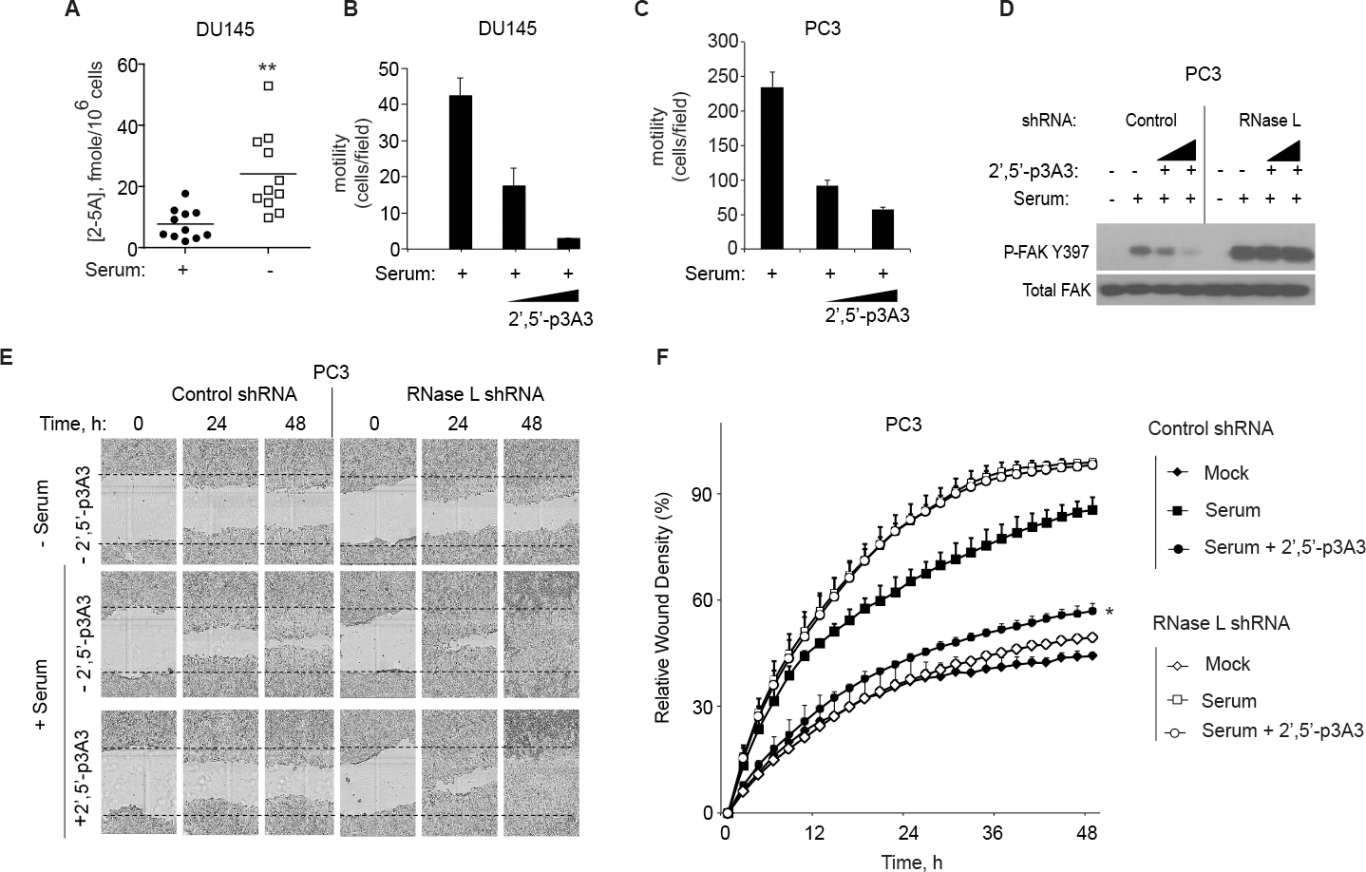
2-5A inhibits cell migration (**A**) DU145 cells were incubated in the presence or absence of serum for 24 h and intracellular 2–5A was measured in cell extracts by FRET assays [28, 29]. Eleven biological replicates with means are shown. (**B, C**) DU145 cells and PC3 cells, respectively, were serum starved for 12 h and transfected with 2–5A[p_3_5′(A2′p5′)_2_A] (100 nM or 200 nM) for 1 h. Cells were trypsinized and transferred into a transwell plate. The assay was done for 5 h with 10% FBS as the attractant and migrating cells were counted and averaged from three wells. Results from 3 technical replicates each are shown. The experiment was conducted 3 times (biological replicates) with similar results. (**D**) FAK phosphorylation after 2–5A transfection in PC3 cells expressing control shRNA or RNase L shRNA by immunoblot assay with monoclonal antibody to FAK(Y397), and Total FAK-monoclonal antibody. (**E, F**) PC3 cells expressing control shRNA or RNase L shRNA were serum starved for 12 h and mock transfected or transfected with 2–5A[p_3_5′(A2′p5′)_2_A] (100 nM) and their migration in absence or presence of serum until 48 h was measured by wound healing assays (as indicated). For each time point, wound closure was measured using Incucyte-ZOOM software. Data are shown as the mean ± SD. The experiment shown includes 6 biological replicates. *p < 0.05, **p < 0.001by Student's two-tailed *t* tests.

To determine intracellular levels of 2–5A, DU145 cell extracts were assayed with a highly specific and sensitive fluorescence energy transfer assay (FRET) [28, 29]. Because cell migration assays are typically performed by adding serum to serum-starved cells, levels of 2–5A were measured after 24 h of serum deprivation and at 24 h after replenishing media with serum (Figure 6A). 2–5A was minimally detectable in serum stimulated DU145 cells. However, after serum starvation, mean intracellular 2–5A levels were increased by about 3-fold. These results suggest that serum deprivation enhances intracellular levels of 2–5A, consistent with a prior study that showed 2–5A accumulated in human T98G neuroblastoma cells during serum starvation [30].

To directly determine if activation of RNase L affects cell migration, transwell assays were done on DU145 and PC3 cells with mock transfection or with transfection of 2–5A for 1 h (Figure 6B, 6C, respectively). 2–5A transfection (100 nM) caused a large decrease in cell migration by 58% and 60% in the DU145 cells and PC3 cells, respectively. Transfection with 200 nM of 2–5A decreased cell migration by 94% and 75% in the DU145 and PC3 cells, respectively.

In addition, in control PC3 cells autophosphorylation at FAK tyrosine-397 occurred in response to serum stimulation and was inhibited by 2–5A transfections. In RNase L deficient cells, serum stimulation also induced FAK phosphorylation at tyrosine-397, but there was no inhibition by 2–5A transfection (Figure 6D). FAK Y397 phosphorylation in response to serum stimulation was enhanced (by 113%) when RNase L levels were depleted when compared to the control cells. Total levels of FAK were unaffected by the presence or absence of RNase L. These data indicate that RNase L renders PC3 cells susceptible to inhibition of FAK autophosphorylation and cell migration in response to 2–5A.

To validate these findings, scratch assays were performed on PC3 cells in the presence or absence of serum and 2–5A transfection. Control and RNase L deficient PC3 cells expressing shRNA were grown in monolayer cultures and then serum starved for 12 h. PC3 control and RNase L deficient cells were transfected with 2–5A [(2′–5′) p_3_A_3_] and monolayers were scratched. The cells were then incubated with 10% serum and time lapse images were captured by Incucyte live cell imaging. Serum caused a 102% and 121% increase in wound closure in the control and RNase L deficient cells, respectively, after 24 h. In control PC3 cells, 2–5A inhibited wound closure by 37% at 24 h of serum stimulation. In contrast, in RNase L deficient PC3 cells, there was no effect of 2–5A on wound closure in the presence of serum. (Figure 6E, 6F).

### RNase L suppresses prostate cancer invasion and metastasis

**Figure 7:**
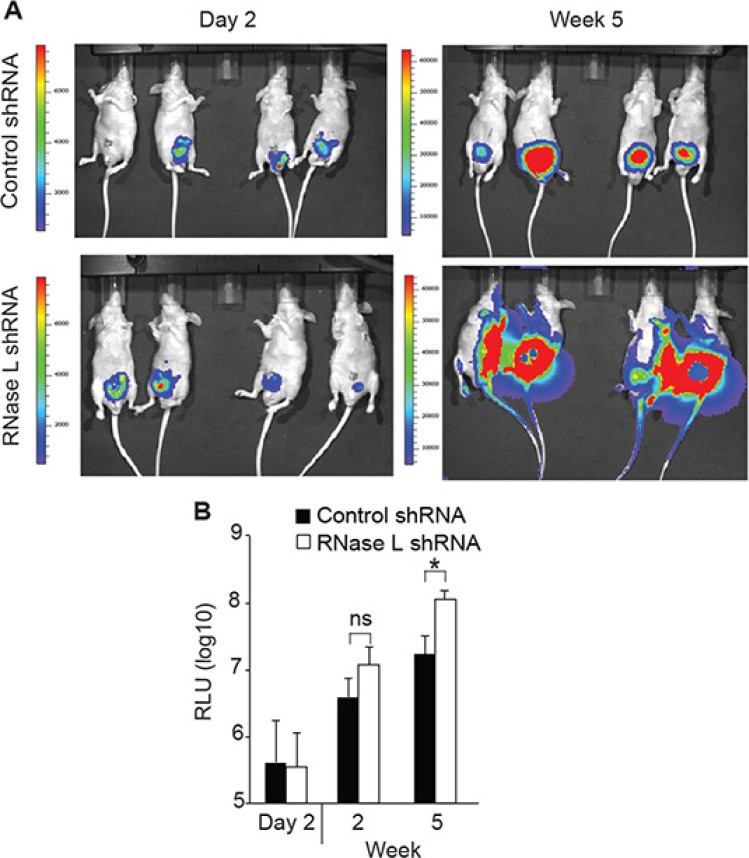
RNase L suppresses tumor growth and metastasis of orthotopically implanted PC3 cells PC3 cells expressing luciferase and vector expressing control shRNA or shRNA against RNase L mRNA were implanted into prostates of nude mice. Tumor growth and metastasis was monitored by real-time imaging. (**A**) Images of mice. (**B**) Relative luciferase units (RLU) (*n* = 8 mice per group). Data are shown as the mean ± SD. ns, not significant; *p < 0.05 by Student's two-tailed *t* tests.

To determine the possible effect of RNase L on metastasis, we used an intra-prostatic implantation model that mimics spontaneous metastasis [31] and examined whether depletion of RNase L in PC3 cells might enhance the metastasis. Luciferase-tagged PC3-control or RNase L-depleted cells were implanted intraprostatically into nude mice and tumor growth in the prostate and its metastases were monitored using bioluminescence. RNase L-depleted PC3 cells implanted in nude mice formed rapidly growing tumors characterized by widespread metastasis compared with the control group by 5 weeks (Figure 7A). In particular, there was metastasis in the fore and hind limbs of the mice implanted with PC3 cells depleted of RNase L, but not in the mice implanted with shRNA control PC3 cells. At 2 week post-implantation, quantification of the tumor sizes based on bioluminescence intensity showed a modest 3-fold [but not significant (*p* = 0.056)] increase in tumor volumes for the RNase L-depleted PC3 tumors compared with the control PC3 tumors (Figure 7B). However, at week 5 the tumor burden of RNase L-depleted PC3 group was significantly greater (> 6-fold; *p* < 0.05) than the tumor burden of the control PC3 group. These data are consistent with our *in vitro* observations, and collectively the results suggests that RNase L inhibits prostate cancer metastasis and that the underlying mechanism might involve RNase L mediated inhibition of cell migration and invasion.

## DISCUSSION

Our findings indicate that RNase L dampens migration of both human and mouse cells. This newly recognized function of RNase L is likely to be relevant to a wide range of different cancer and normal cell types. Inhibition of cell migration was greatly increased upon 2–5A activation of RNase L, which often occurs when cells are infected by viruses [2], possibly restricting viral spread within an organism. We show here that serum starvation induced low levels of intracellular 2–5A. RNase L is activated by subnanomolar levels of 2–5A [20, 29], therefore even low levels could be functionally significant. Similarly, serum-deprivation of human neuroblastoma T98G cells was previously shown to induce accumulation of intracellular 2–5A [30]. The reason why serum starvation leads to increased levels of 2–5A is unknown, but could possibly be due to induction of OAS and/or its RNA activators.

It appears that the catalytic domain of RNase L is required for optimal inhibition of cell migration because the R667A mutation in RNase L, which causes a complete loss of ribonuclease activity ([26] and Figure 5), has no apparent effect on cell migration in PC3 cells. While protein-protein interaction might contribute to the effect, interaction of RNase L with actin binding protein filamin A seems unlikely to be involved because both wild type RNase L and mutant R667A RNase L bind filamin A [32]. However, in mouse MEF, catalytically inactive mutant RNase L W630A reduced migration by about 2-fold, although this was less than the 3.5-fold inhibition obtained with WT RNase L. In contrast to our findings, a prior study reported that RNase L deficiency reduced, rather than increased, the migration of mouse bone marrow macrophages (BMM) in response to serum stimulation [33]. Therefore, the regulation of cell migration by RNase L displays suggestions of cell type differences. Our findings suggest that both catalytic and non-catalytic domains of RNase L have anti-cell migration properties in mouse MEF. However, in PC3 cells a functional catalytic domain of RNase L was essential for suppression of cell migration.

The precise mechanism by which RNase L suppresses cell migration remains to be determined. While RNA cleavage products generated by RNase L were previously shown to stimulate signaling through RIG-I like receptors, depletion of RIG-I, MDA5 or MAVS in PC3 cells by means of siRNA did not affect cell migration (data not shown). Therefore, cleavage of mRNAs for proteins involved in cell adhesion and migration appears a more likely mechanism for the inhibition of cell migration by RNase L. Our results extend the known biologic roles of RNase L to inhibition of cell migration offering a possible explanation for the proposed link between mutations or variants in the RNase L gene and cancer risk [3–6, 11–13].

## MATERIALS AND METHODS

### Cell Culture

WT and *Rnasel*−/− MEFs cell lines transformed with SV40 T antigen [15], DU145, and PC3 cell lines (ATCC) were grown in RPMI 1640 cells with 10% FBS and antibiotics. PC3 expressing luciferase cDNA [from pLentiCMVV5-LUC Blast (w567–1)] were used for all experiments involving PC3 cells (referred to in the text as “PC3”). RNase L shRNA expressing PC3 cells, and the corresponding control shRNA expressing cells, were maintained in media with blasticidine (1μg/ml) and puromycin (2.5 μg/ml). The PC3 cells expressing RNase L shRNA, empty vector, WT or mutant human RNase L (R667A) were maintained in media with blasticidine (1 μg/ml), puromycin (2.5 μg/ml) and neomycin (500 μg/ml). 293T cells were grown in DMEM supplement with 10% FBS. NIH3T3 cells and the derivative cell line pLZ NIH3T3 overexpressing human RNase L [27] were grown in DMEM with 10% FBS.

### Cloning of mutant mouse RNase L W630A

Mouse RNase L W630A mutant was constructed from WT mouse RNase L cDNA (NCBI Reference Sequence:NM_011882.2) in vector p3 × Flag CMV-10 (purchased from Sigma-Aldrich) using the Modified Megaprimer PCR method. Briefly, a 360 bp megaprimer was generated in the first round of PCR (PhusionHiFi PCR kit, New England Biolabs) incorporating the mutagenic primer. The megaprimer and two flanking cloning primers were used in a subsequent round of PCR to produce the full length 2.2 Kb mutagenic insert which was ligated into p3 × Flag CMV-10. The construct insert was fully sequence verified.

### Construction of pseudolentivirus for CRISPR/Cas9 knockout of the human RNase L gene

The oligonucleotide sequence (5′- tttgaggcgaaagacaaagg-3′) for generation of small guide RNA (sgRNA) targeting the first exon of RNase L was selected from a published sgRNA database [34]. The lentiviral particles were prepared as described [35]. Briefly, a pair of oligonucleotides were synthesized and annealed, the plasmid Lenti-CRISPR (Addgene) was digested by BsmBI (NEB), the DNA fragments were inserted into the vector to generated plenti-CRIPSR-sgRL-6. The pseudo lentiviruses were packaged in 293T cells. Briefly, 1 × 10^6^ cells were plated in one well of 6-well plate, the next day cells were transfected with 5 μg of plenti-CRIPSR-sgRL-6, 3.5 μg psPAX2 and 1.25 μg of pCMV-VSV-G (a gift from Paul Bates, University of Pennsylvania) by Lipofectamine 2000 (24 μl in 250 μl of DMEM). The supernatants were harvested at 24 and 48 h post transfection, and were stored at −80°C.

### Antibodies

Phospho-FAK (Y397) and total FAK antibodies were from Cell Signaling Technology, anti-mouse Flag antibody and β-actin antibody were from Sigma-Aldrich. Blocking anti-integrin β1 antibody (ab24693) or anti-integrin alpha V β3 antibody (ab78289) were from Abcam, and anti-human RNase L monoclonal antibody was as we described [36].

### Transfections

All transfections (except with siRNAs) were done with Lipofectamine 2000 (Invitrogen) according to the manufacturer's protocol. siRNA transfections were done with Dharmafect1 (Thermo Scientific, LifeTechnologies). DU145 cells (2.5 × 10^5^ cells) were transfected with pre-made *SMART* pool siRNA targeting RNase L (Santa Cruz) using Dharmafect1 for 48 h. 2–5A (2′, 5′-p_3_A_3_, prepared as described [21]), was transfected with Lipofectamine 2000.

### Lentiviral shRNA-based knockdown of gene expression

Lentivirus expressing control shRNA and shRNA targeting the 3′-UTR of RNase L (GenBank NM_021133.2-2410s21c1, TRCN0000226437) were described previously [17, 37]. Hek293T cells were transfected with (pCMV-VSV-G, expressing vesicular stomatitis virus G envelope protein, and the pCMV-dR8.2 packaging plasmid, from Robert Weinberg [38]) along with shRNA expressing plasmids using Lipofectamine 2000. Virus-containing medium was collected 48 h after transfection and used immediately. PC3 cells expressing luciferase were infected at a density of 1 × 10^6^ cells ml^−1^ in the presence of 8 μg/ml polybrene, overnight. The stable cells were selected with puromycin (2.5 μg/ml). 3 × FLAG-human RNase L or human mutant RNase L R667A was PCR amplified to be inserted in lentiviral vector pCMV-PL4-Neo (Addgene, Principal Investigator Eric Campeau) as described previously [17] and the lentivirus were prepared as described above. Virus-containing medium was collected 48 h after transfection and used immediately. PC3 cells were infected at a density of 10^6^ cells/ml in the presence of 8 μg/ml polybrene, overnight. The stable cells were selected with neomycin (G418) (500 μg/ml).

### Transwell cell migration assays

Migration of cells was assessed by using 6.5 mm Transwell^®^ with 8.0 μm Pore Polycarbonate Membrane Insert (Sterile) (Corning LifeSciences) and media containing 10% FBS or fibronectin (10 μg/ml) in the lower chamber as the attractant. Cells (2 × 10^4^) suspended in media containing bovine serum albumin (BSA) (Sigma-Aldrich) (200 μg/ml) were seeded into each of six wells of the upper chamber. The cells were allowed to migrate in transwell assays for 4 h at 37°C, and then adherent cells on the upper surface of the membrane were removed by scraping. Migratory cells attached to the bottom of the membrane were fixed and stained with 0.1% crystal violet. Images of migrating cells were captured by using an inverted phase contrast (EVOS-Life Technologies). For each cell type, three independent experiments were performed.

### Monolayer scratch-wound healing assay

Unless stated otherwise, wound healing assays were done by seeding cells in 96-well plates (Essen ImageLock, Essen Instruments), and incubating in serum free medium for 24 h [39]. After 16 h, wounds were made by scratching with wound scratcher (Essen Instruments). Cells were stimulated with 10% serum immediately after wound scratching, and wound confluence was monitored with Incucyte Live-Cell Imaging System and software (Essen Instruments 2015 A). Wound closure was observed every hour for indicated times by comparing the mean relative wound density of at least four biological replicates in each experiment [39]. For some experiments, manual wound healing assay were performed. Briefly, confluent cell cultures were grown in 6-well plates and incubated 24 h in serum-free medium. Monolayers were scratched with a micropipette tip [40]. Cells were washed with warm PBS, and maintained in media with 10% FBS. To analyze cell migration, the plates were placed into a temperature- and CO_2_-controlled incubator (at 37°C, 5% CO_2_) on the stage of a Leica DMIRE2 inverted microscope (Leica, Bannockburn, IL), equipped with electronically controlled shutters and filter wheel. Phase contrast images were captured under a 10 × objective with a camera controller C4742_95 (Hamamatsu, Bridgewater, NJ) and run by ImprovisionOpenlab software, version 3.1.5, and every 5 min for 24 h. The area occupied by migration cells was calculated from each image in NIH-image J with Graph Pad Prism software. At least three independent experiments were carried out for each experimental condition.

### Ribosomal RNA (rRNA) cleavage assays

rRNA cleavage was monitored as we described previously [41]. Briefly cells were transfected with poly(I):poly(C) (pIC) with Lipofectamine 2000 and after 5 h the total RNA was isolated using TRIzol (Invitrogen) and resolved on RNA chips using an Agilent 2100 BioAnalyzer.

### Fibronectin replating and FAK phosphorylation

Fibronectin replating experiments with WT and *Rnasel*−/− MEF were performed as described [42], except 10 μg of fibronectin (Sigma-Aldrich)/ml was used. FAK phosphorylation was detected by Western blot analysis using anti-phospho FAK (Y397) antibody.

### Immunoblotting

Prior to lysis, cells were washed twice in cold phosphate-buffered saline (PBS). Cell extracts were prepared with lysis buffer supplemented with phosphatase/protease inhibitors followed by incubation on ice for 20 min. Lysates were subjected to centrifugation at 12,000 *g* for 10 min, and the supernatants collected and protein quantified by Bradford assays (BioRaD). Cell lysates (30–50 μg) were separated on 8% or 10% SDS PAGE gels and proteins were transferred to polyvinylidenedifluoride membranes (0.45 μm) (BioRaD) and probed with antibodies according to the different manufacturers' recommendations.

### Orthotopic mouse prostate tumor model

PC3 cells expressing luciferase (10^6^ cells in a total volume of 0.02 ml) were inoculated into the prostate of 8- to 12- week-old athymic nude mice (*n* = 8 mice for each group) through an abdominal incision [31]. For pain control, buprenorphine (0.05 mg/kg i.m.) was given immediately preoperatively. Anaesthetic consisted of ketamine/xylazine/acepromazine (50/5/1 mg/kg) given i.p. in a volume of 0.1 ml. Skin was prepared with betadine followed by ethanol wipe. Bupivicaine was injected (0.1 ml) in the area of the skin incision to provide pain relief. Sterile drapes, sterile instruments, and sterile gloves were used. An incision 4 mm long was made over the bladder with a scalpel. Tumor cells in a volume of 20 μl were inoculated into the ventral lobe of the prostate gland. Muscle layer was closed with 5–0 absorbable suture. Skin was closed with a stainless steel clip. *In vivo* bioluminescent imaging was done using an IVIS Xenogen pre-clinical imaging system at 2 days and at every 7 days after implantation for 6 weeks. Mice were transferred to the induction chamber containing an isoflurane (1–3%) /oxygen mixture which was also used during imaging. Mice received luciferin (3 mg delivered i.p.). Anesthetized mice were transferred to the imaging chamber where they were imaged for 5 min. Animal experiments using male NCr-nu/nu mice were performed in accordance with recommendations in Guide for the Care and Use of Laboratory Animals of the National Institutes of Health, and conducted under a protocol approved by Cleveland Clinic Institutional Animal Care and Use Committee.
